# Web Search Trends of Implementing the Patient Autonomy Act in Taiwan

**DOI:** 10.3390/healthcare8030353

**Published:** 2020-09-21

**Authors:** Po-Chin Yang, Mei-Ju Shih, Ya-An Liu, Ya-Chuan Hsu, Hsiao-Ting Chang, Ming-Hwai Lin, Tzeng-Ji Chen, Li-Fang Chou, Shinn-Jang Hwang

**Affiliations:** 1 Department of Family Medicine, Taipei Veterans General Hospital, Taipei 112, Taiwan; michael00557@gmail.com (P.-C.Y.); a3786923@gmail.com (Y.-A.L.); ych97160@gmail.com (Y.-C.H.); htchang2@vghtpe.gov.tw (H.-T.C.); tjchen@vghtpe.gov.tw (T.-J.C.); sjhwang@vghtpe.gov.tw (S.-J.H.); 2 School of Medicine, National Yang-Ming University, Taipei 112, Taiwan; 3 Graduate Institute of Communication Engineering, National Taiwan University, Taipei 106, Taiwan; b96901063@ntu.edu.tw; 4 Department of Public Finance, National Chengchi University, Taipei 116, Taiwan; lifang@nccu.edu.tw

**Keywords:** advance care planning, google trends, patient autonomy act, Taiwan

## Abstract

Background: The Patient Autonomy Act was implemented in Taiwan on 6 January 2019. It is the first patient-oriented act in Taiwan, and also the first special act to completely protect patient autonomy in Asia. Our study aimed to investigate the web resources citizens were able to access on the eve of the implementation of the Patient Autonomy Act in Taiwan. Methods: Patient Autonomy Act-related web resources were searched for by entering 10 related terms individually into the Google search engine in January 2019 and again in April 2019. Search activity data were analyzed using Google Trends. Results: “Advance care planning” and “advance decision” were the most relevant keywords for finding information about the Patient Autonomy Act on the eve of the act’s implementation in Taiwan. The main online information sources were non-governmental websites including news sites and online magazines. The related search volume only increased on the eve of implementation. Conclusions: Even though the Patient Autonomy Act was first published three years before its implementation, the related search volume only increased on the eve of its implementation. Therefore, whether the three-year buffer between its publication and implementation was necessary requires further investigation.

## 1. Introduction

### 1.1. Advance Care Planning (ACP) and Patient Autonomy Act in Taiwan

Advance care planning (ACP) is a process in which a person makes decisions for themselves about their own future medical treatment after consulting with healthcare professionals [1]. It enables care providers to better understand and respect a person’s preferences in the event that the person becomes critically ill and unable to speak for themselves. A complete advance care plan covers the key issues relating to life and death, including the goal of care for the patient, the use or non-use of life support and resuscitation efforts, the designated surrogates for decision making, and the completion of advance directives (ADs). Team-based frameworks rather than physician-led approaches are required for appropriate ACP [2]. The general awareness and accessibility of ACP have increased in the past few decades in various countries. ACP is supported by legislation in Australia, the United Kingdom, and the United States [3]. Several organizations aimed at offering ACP support have also been founded in the United States, Canada, Australia, and New Zealand [2]. Previous studies have concluded that ACP has some positive impacts on end-of-life care, such as better quality of life and decreased hospital admission rates [4]. Another study revealed that ACP can improve patient and family satisfaction, in addition to lowering stress, anxiety, and depression in surviving relatives [3].

In Taiwan, a new law pertaining to ACP, the Patient Autonomy Act, was first published on 6 January 2016, before coming into force three years later on 6 January 2019. It is the first patient-oriented act in Taiwan, and also the first special act to completely protect patient autonomy in Asia. The Patient Autonomy Act is relevant to the welfare of all citizens and healthcare providers. However, even three years after the act’s publication and subsequent government efforts to publicize it, there were still some citizens were who were unfamiliar with the act and misunderstood various topics related to it. For example, some people even confuse ADs with assisted suicide or euthanasia. So far, we can still see many patients undergoing unnecessary cardiopulmonary resuscitation or intubation before the end of life. Ideally, with a full understanding of the Patient Autonomy Act, one can complete his/her own ADs, with the ultimate goal being to achieve a “peaceful death” according to his/her own wishes.

### 1.2. Health-Related Information on the Internet

The Internet developed rapidly over the past several decades, becoming the primary means by which the public searches for information. The Google search engine is one of the tools most commonly used by Internet users to access health-related information [5]. One study showed that 80% of Internet users use the Internet to access healthcare information [6]. Meanwhile, another study found that in 2015, 5% of 1.2 trillion Google searches were for health-related information [7]. However, the quality and readability of search results vary, and whether or not a website that an Internet user visits is produced by a credible organization and offers clear and reliable information is often unclear. For example, one previous study revealed that most of the palliative care-related education articles available via the Google search engine were written at levels above national health literacy recommendations, meaning that they were too advanced for most users to understand [8]. Another study focused on the online palliative care resources accessible via the Google search engine and concluded that the search results were mostly consistent with the definition of palliative care at present [9]. Interestingly, about 92% of Internet users only view the first 10 search results [10], with the information from larger organizations or from government agencies generally being considered more credible than information from other sources.

Taiwan has a high Internet penetration rate. According to a survey conducted by the Taiwan Network Information Center in 2019, the Internet penetration rate reached 85.6% [11]. The high Internet penetration rate altered citizen’s lifestyles. It was also reported that people largely rely on the Internet for instant communication, news, live video, mail, social media, etc. Another statistic study conducted by the StatCounter Global Stats showed that Google accounted for 92.87% of all search engine usages in Taiwan in August 2020 [12]. The number was 87.74% in June 2018 [13]. We can see that the Google search engine is the mainstream search engine that is broadly used in Taiwan, and the analysis of the Google search engine is compatible with the general trends in Taiwan.

### 1.3. Google Trends and Health

Google Trends (GT; Google, Mountain View, CA, USA) is a free and publicly available online tool that users can utilize to graph the popularity of one or more terms over a period of time [14]. For a given term, it provides users with the related search volume (RSV), with the fluctuations in the RSV reflecting Internet users’ changing search interests over time. The tool can also show search volumes for different geographic regions, as well as those for related search terms. An RSV = 100 is defined as the peak search volume over a specific period of time. The search volumes at other time points are presented on a scale from 0 to 100, which may reflect the dynamic activity of each term over time. GT can provide either a daily or weekly RSV depending on the duration of the search timeframe. In light of these capacities, GT (or Google Insights, the previous similar Google tool) has previously been used in numerous health-related research studies, such as studies engaged in real-time tracking of infectious diseases like Dengue fever [15], Lyme disease [16], and the RSV virus [17], as well as studies aimed at predicting disease outbreaks [18]. It has also been applied in studies regarding several non-infectious diseases and health issues associated with non-infectious diseases, including cancer screening [19], dementia [20], tobacco use [21], suicide risk evaluations [22], and women’s health [23].

This study aimed to investigate the web resources that citizens in Taiwan were able to access on the eve of the implementation of the Patient Autonomy Act. We focused on the search results provided by the Google search engine for various related terms, as well as on the search volumes for those terms indicated by Google Trends. The results of our study can in turn be used to provide the citizens of Taiwan with a more accurate understanding of the Patient Autonomy Act and might facilitate international comparison, and provide health policymakers or administrators with insights regarding the allocation of resources for strengthening the functions of Internet websites, in order to promote more accurate information for people.

## 2. Materials and Methods

### 2.1. Patient Autonomy Act-Related Terms Internet Search

All of the data used in this study were publicly available online. We analyzed Patient Autonomy Act-related web resources by individually entering 10 terms (in Chinese) related to the Patient Autonomy Act into the Google search engine. The first 50 search results were recorded and then classified according to whether the content was related to the Patient Autonomy Act of Taiwan, which was first published on 6 January 2016, and whether the source was from a credible organization. The 10 search terms used were “advance care planning”, “advance decision”, “advance directive”, “hospice palliative care”, “refuse treatment”, “do not resuscitate”, “extubation”, “withdraw life-sustaining treatments”, “suicide assistance”, and “euthanasia”. The search result resources were classified into the following categories: Health and Welfare Department and its affiliated organizations (.mohw.gov.tw), other public hospitals and local health institutions (.gov.tw), private hospitals (.org.tw∩ hospitals), non-governmental organizations (.org.tw∩ organizations), non-governmental websites, electronic news or electronic magazines, blogs (pixnet.net∪blogspot.com), Facebook pages, YouTube pages, explanations of laws and regulations from law firms, patient autonomy research centers (.parc.tw), and Wikipedia (wikidepia.org).

There were two stages for our Internet web searches. The first stage of searches was conducted on 5 January 2019, the eve of the implementation of the Patient Autonomy Act in Taiwan. The second stage was conducted on 5 April 2019, three months after the implementation of the Patient Autonomy Act. The differences between the results for the two stages were then compared.

### 2.2. Patient Autonomy Act-Related Terms on Google Trends

We conducted a retrospective analysis of Patient Autonomy Act-related web search activity data by using Google Trends (http://www.google.com/trends). We entered related search terms (in Chinese) into the Google Trends main page for web searches. Our research covered the time between 6 January 2016 (when the Patient Autonomy Act was first published), and 5 April 2019 (three months after the implementation of the Patient Autonomy Act), and limited the search region to Taiwan only, with all categories of search. The search terms used included “Patient Autonomy Act”, “advance care planning”, “advance decision”, “advance directive”, “hospice”, “hospice ward”, “palliative care”, “do not resuscitate”, “DNR”, “extubation”, and “euthanasia”. We downloaded raw data as CSV datasets and analyzed the RSV results, which reflected the dynamic activity of each search term over time. Weekly data was used in our study. We then compared different combinations and tried to interpret the tendencies we observed.

## 3. Results

### 3.1. Internet Searches

Based on the first 50 results for each of the 10 search terms related to the Patient Autonomy Act when we searched using the Google search engine on 5 January 2019, we found that the terms “advance care planning” and “advance decision” had the strongest relationship with the Patient Autonomy Act, with all of the results (50/50) for each term being related to the act. Otherwise, 54% (27/50) and 28% (14/50) of the results for the terms “advance directive” and “refuse treatment”, respectively, were related to the act. The other terms had only a few (<10%) or no related search results (Table 1).

As for the sources of the search results, the main sources were non-governmental websites including news sites and online magazines, which accounted for 38.6% (56/145) of all the search results. Private hospitals and non-governmental organizations accounted for 31.0% (45/145) of the results, while the Health and Welfare Department and its affiliated organizations and other public hospitals and local health care institutions accounted for 17.2% (25/145) of the search results. Other sources accounted for only a few search results (Table 1).

Based on the first 50 results for each of the 10 search terms related to the Patient Autonomy Act when we searched using the Google search engine on 5 April 2019, the terms “advance care planning” and “advance decision” still had the strongest relationship with the Patient Autonomy Act, with all of the results (50/50) for each term being related to the act. Otherwise, 62% (31/50) and 44% (22/50) of the results for the terms “advance directive” and “refuse treatment”, respectively, were related to the act. The other terms still had only a few (<10%) or no related search results (Table 2).

As for the sources of the search results, non-governmental websites including news sites and online magazines were still the main sources, accounting for 39.5% (62/157) of all the search results. Private hospitals and non-governmental organizations accounted for 29.3% (46/157) of the search results, while the Health and Welfare Department and its affiliated organizations and other public hospitals and local health care institutions accounted for 21.0% (33/157) of the search results. Other sources accounted for only a few or none of the search results (Table 2). There were no significant differences between the results for the two different search dates.

### 3.2. Google Trends

**Figure 1:**
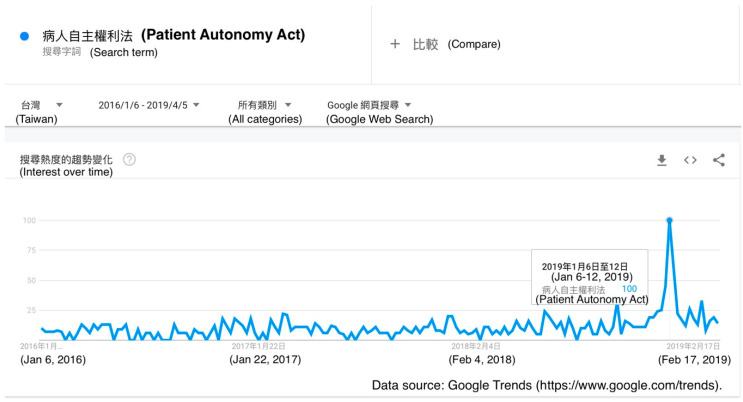
A Google Trends graph for the term “Patient Autonomy Act” (blue line) in Taiwan from 6 January 2016 to 5 April 2019.

The Google Trends graph for the term “Patient Autonomy Act” showed a peak in January 2019, around the implementation date of the act. There were no other significant search peaks since January 2016 (Figure 1).

**Figure 2:**
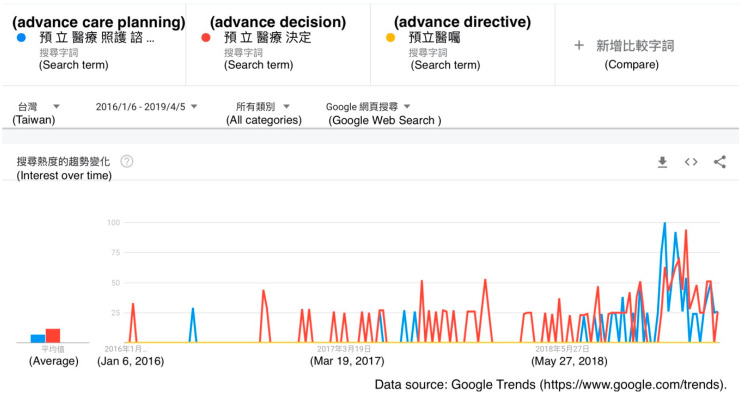
A Google Trends graph for the terms “advance care planning” (blue line), “advance decision” (red line), and “advance directive” (yellow line) in Taiwan from 6 January 2016 to 5 April 2019.

The Google Trends graph for the terms “advance care planning”, “advance decision”, and “advance directive” also showed a peak for “advance care planning” and “advance decision” in January 2019, around the implementation date of the act. There were no other significant search peaks since January 2016. There was not a substantial volume of searches using the term “advance directive” in comparison to the volumes of searches using the other two terms (Figure 2).

**Figure 3:**
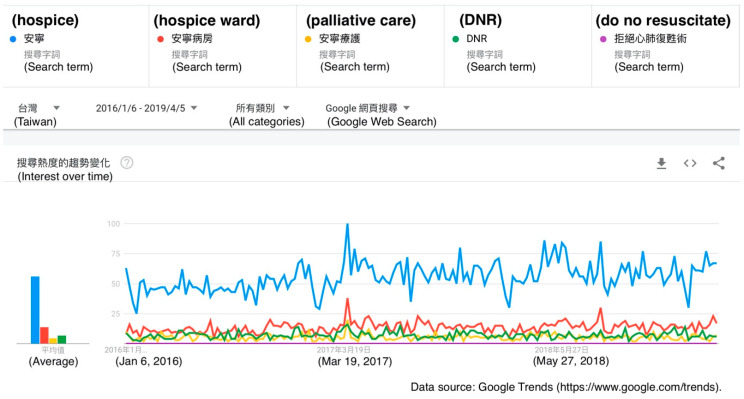
A Google Trends graph for the terms “hospice” (blue line), “hospice ward” (red line), “palliative care” (yellow line), “do not resuscitate” (purple line), and “DNR” (green line) in Taiwan from 6 January 2016 to 5 April 2019.

The Google Trends graph for the term “hospice” showed a higher volume of searches than the terms “hospice ward”, “palliative care”, “do not resuscitate”, and “DNR”. There was a relative peak in searches using these terms around March 2017. There was not a substantial volume of searches using the term “do not resuscitate” in comparison to the other terms, but there are notable volumes of searches using its abbreviation “DNR” (Figure 3).

**Figure 4:**
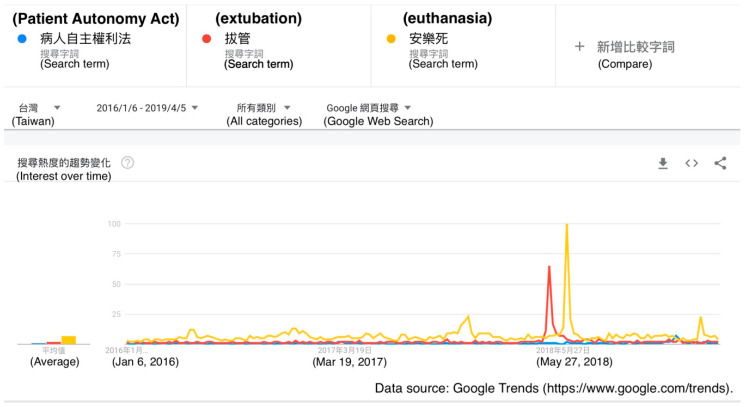
A Google Trends graph for the terms “Patient Autonomy Act” (blue line), “extubation” (red line), and “euthanasia” (yellow line) in Taiwan from 6 January 2016 to 5 April 2019.

In comparison to the term “Patient Autonomy Act”, there was a significant peak in the volume of searches using the term “extubation” around April 2018, and a significant peak in the volume of searches using the term “euthanasia” around June 2018. There were no significant differences among the terms at any other time points (Figure 4).

## 4. Discussion

### 4.1. ACP in Taiwan

Taiwan’s Hospice Palliative Care Act was first legislated in 2000 and has since been amended three times, with the latest amendment occurring in January 2013. Taiwan’s Hospice Palliative Care Act respects the self-determination of patients with terminal illnesses and protects their rights with respect to the non-application of certain forms of medical management, such as cardiopulmonary resuscitation (CPR) and other forms of life-sustaining treatment (LST), through written ADs [24]. As stated in Article 7 of the act, the act becomes effective when a patient has been diagnosed with a terminal illness by two physicians and has signed a letter of intent. A close relative of the patient may sign a consent in lieu of the unconscious patient if the patient has failed to express his/her own will [25]. However, some constitutional concerns are still noted under some special circumstances [26]. Furthermore, a patient’s “do not resuscitate” (DNR) request can be written on his or her National Health Insurance IC Card since 2006 [27].

In contrast with the previous Hospice Palliative Care Act that limited the certain choices to terminally ill patients, the Patient Autonomy Act allows all patients to establish ADs to decide what kinds of LSTs and artificial nutrition and hydration they would refuse to receive in advance. The conditions invoking such ADs were also expanded to five clinical conditions including terminal illness, irreversible coma, permanent vegetative state, severe dementia, and other disease conditions in which the disease is incurable and there are no other appropriate treatment options available given the medical standards [25].

Through the implementation of the Patient Autonomy Act, everyone is able to engage in introspection regarding the value of life and then decide about his/her own medical care according to his/her own preferences. Also, everyone can assign his/her most trusted surrogate to ensure that an AD is followed, with that surrogate is not limited to his/her relatives. Through a comprehensive advance care plan, one can receive consultation regarding the content of an AD. After gaining a full understanding of the different choices, including the pros and cons of each option, one can complete his/her own ADs, with the ultimate goal being to achieve a “peaceful death”.

### 4.2. Patient Autonomy Act-Related Web Sources

In our study, we found that in addition to direct searches with Internet search engines using the keyword “Patient Autonomy Act”, the terms “advance care planning” and “advance decision” were the most relevant keywords for finding Patient Autonomy Act-related information on the web. We focused on the first 50 search results for each keyword because most users do not have enough patience to continue searching if they cannot find the information they need after viewing the first few pages of search results. Interestingly, one study showed that about 92% of Internet users only view the first 10 search results [10]. Thus, there is a certain art to finding the information one needs.

We assume that curious users may search using different keywords to find the information they need. However, by using some keywords that do not have exactly the same definitions, one may sometimes be unable to find accurate results. The terms “advance care planning” and “advance decision” were directly related to the Patient Autonomy Act. The Patient Autonomy Act protects one’s right to attend to ACP and try to sign an AD. As for our other search terms, the term “advance directives” had a wider range of results than the term “advance decision”, with the results for the former not being limited to the specific form one fills out after ACP. It usually refers to the letter of intent for the choice of hospice palliative care or LST and the agreement regarding a DNR request. Hospice palliative care is aimed at relieving suffering and improving the quality of life for persons with advanced illnesses. The term “refuse treatment” refers to refusing any kinds of medical treatments beyond LST and artificial nutrition and hydration. The term “do not resuscitate” is focused on the actions of cardiac percussion, endotracheal tube insertion, electronic defibrillation, and the use of first-aid medicine. The terms “extubation” and “withdraw life-sustaining treatments” are specific to requests to stop certain life-sustaining treatments that may already be in use. Assisted suicide and euthanasia are not legal in Taiwan, as the essence of the Patient Autonomy Act is not to prolong a patient’s time until an unavoidable death, but also not to accelerate such a death. Nonetheless, some people may have some confusion regarding the terms “suicide assistance” and “euthanasia”, and some websites seek to clarify the meanings of these terms.

Our study also found that non-governmental websites including online news sites and online magazines were the main source of information on the Internet by using the 10 investigated search terms. Generally, the information from larger organizations or from government agencies was considered more credible information. In the era of the Internet, the content of some websites is refreshed every day, and people in many countries can set up their own websites freely. As such, the contents of many sites on the web may not be true. We should thus judge the accuracy of information carefully to avoid any misunderstandings. We suggest for health policymakers or administrators devoted to making more clear and understandable webpages to promote accurate information for people. Furthermore, they should also try to make the information easily accessible. For example, make it easier to be found by search engines, or be broadly linked through social media. By doing so, people can acquire correct healthcare knowledge more easily, which can in turn enhance public health.

### 4.3. The Popularity of Patient Autonomy Act-Related Searches

The use of the Internet to access information on the Patient Autonomy Act in Taiwan appears to have been relatively limited in Taiwan at the time when the act was established in January 2016. However, when the act was about to be implemented, the volume of searches regarding the act increased, peaking around January 2019. Also, the related terms “advance care planning”, “advance decision”, and “advance directive” had similar trends in that the associated search volumes increased only at the time of the act’s implementation. The goal of the three-year buffer between the act’s publication and its implementation was to make citizens and care providers more prepared for the changes brought by the act. However, we can observe that the popularity of searches for related concepts was generally low after the act was first established, only peaking when the act was about to be implemented. As such, whether the three-year buffer between the act’s publication and implementation was necessary requires further discussion and evaluation.

We also found that the term “hospice” was generally more searched for than other terms such as “hospice ward”, “palliative care”, “do not resuscitate”, and “DNR”. Taiwan’s first hospice ward was built in 1990. Taiwan’s Hospice Palliative Care Act was first established in 2000 and has since been amended three times, with the aim of respecting terminally ill patients’ will regarding medical treatments and protecting their associated rights. As hospice home care and hospice admission were included into the National Health Insurance system, the acceptance of palliative care among the citizens of Taiwan gradually increased, and now there are 76 hospice wards in Taiwan, distributed in all cities and counties, providing 837 beds for patients in need [28]. According to the ratings of terminally ill patients’ quality of death announced by the Economist Intelligence Unit in 2015, Taiwan ranked number six among all 80 countries/territories included in the ratings and had the best rating in Asia [29]. Thus, people in Taiwan have gradually become more willing to receive palliative care. The Patient Autonomy Act has further enlarged the scope of the application of such care, allowing all patients to establish ADs in advance. The goal of a “good death” will thus generally be easier to achieve under the protection of the Patient Autonomy Act.

### 4.4. The Popularity of Various Searches in Relation to Current Events

We also found the interesting result that the popularity of certain searches may reflect certain events that happened at a given time. For example, the peak in searches using the term “hospice” was in March 2017, when a famous fiction writer named Qiong Yao wrote a public letter to her son and daughter-in-law on Facebook about her attitude toward death and her wish not to receive any major operations, not to be admitted to an intensive care unit, and not to undergo NG tube insertion once she became critically ill [30]. That letter induced a lot of discussion and made people more curious about the idea of palliative care.

Also, searches using the term “extubation” peaked around April 2018, at the time when the president of the National Taiwan University was replaced. This was because the last name of the president who was replaced was “Guan”, which also means “tube” in Chinese, while “extubation” means “to remove the tube”. The news caused a mighty uproar at the time, and thus “extubation” was a popular search term that people were curious about. Searches using the term “euthanasia” had a peak in June 2018, when a famous sports anchor named Frank Fu went to Switzerland to undergo euthanasia at the age of 85 after suffering from terminal pancreatic cancer. Euthanasia is still not legal in Taiwan, and his choice dominated the news at the time. Then, his son committed to efforts to legalize euthanasia and tried to raise public awareness of dying with dignity. These examples reveal that current events influence search trends and even affect people’s cognitions and values on certain issues. Social media should seek to uphold objective neutrality and deliver accurate facts to the public.

### 4.5. Limitations

There were some limitations to our study. First, the web-page survey part was a cross-sectional study in nature. We recorded the content we found by using certain keywords at two different time points (January 2016 and January 2019) and then compared the differences in the results, but we cannot know the dynamic changes in the search results. Second, we used Google Trends to analyze the dynamic search volumes, but GT only offers relative search volumes but not absolute numbers of searches. Thus, the real popularity of the investigated keywords remains unknown. The question of exactly how many Internet users were interested in related topics thus requires more investigation. Third, we only focused on the related terms we thought to be commonly used after our team discussion, and some keywords may have been neglected. Fourth, relatively younger people are inclined to get information from web searches, but older people do not. Not all generations in Taiwan may use Google web search as their main information resource, further investigation is needed to clarify the use of Google web search in different generations. Finally, Internet-use habits differ from region to region, so our study results cannot be taken as representative of all the Internet search patterns around the world.

Our future work will be trying to enhance the awareness of the Patient Autonomy Act. Thus, more comprehensive methodologies for Internet information extraction should be used to enhance website content analysis process efficiency. We will try to strengthen the functions of these Internet searching tools, in order to promote more accurate information for people.

## 5. Conclusions

On the eve of the implementation of the Patient Autonomy Act in Taiwan, the terms “advance care planning” and “advance decision” were the most relevant keywords for finding related information on the act. The main websites providing such information were non-governmental websites including online news sites and online magazines.

However, even though the Patient Autonomy Act was first published three years before its implementation, the related search volume only increased on the eve of its implementation. Therefore, whether the three-year buffer between its publication and implementation was necessary requires further investigation.

## Figures and Tables

**Table 1:** The number of search results for each of the different terms that were related to the Patient Autonomy Act when searching with the Google search engine on 5 January 2019.

Category of Search Results	Root Domain	Advance Care Planning	Advance Decision	Advance Directive	Hospice Palliative Care	Refuse Treatment	Do Not Resuscitate	Extubation	Withdraw Life-Sustaining Treatments	Suicide Assistance	Euthanasia
Health and Welfare Department and its affiliated organizations	.mohw.gov.tw	2	2	1	0	0	0	0	0	0	0
other public hospitals and local health care institutions	.gov.tw	9	4	6	0	0	0	0	1	0	0
private hospitals	.org.tw∩ hospital	11	11	5	0	0	0	0	0	0	0
non-governmental organizations	.org.tw∩ organization	8	7	1	0	1	0	0	1	0	0
non-governmental websites		9	12	4	0	4	0	0	1	0	0
electronic news or electronic magazines		5	8	8	0	4	0	0	0	0	1
Blogs	pixnet.net ∪ blogspot.com	0	0	0	0	1	0	0	0	0	0
Facebook pages		0	0	0	0	0	0	0	0	0	0
YouTube pages		1	1	0	0	2	0	0	0	0	0
explanations of laws and regulations from law firms		2	3	1	0	0	0	0	0	0	0
patient autonomy research centers	.parc.tw	1	1	1	0	2	0	0	0	0	0
Wikipedia	wikipedia.org	1	1	0	0	0	0	0	0	0	0
Total		50/50 (100%)	50/50 (100%)	27/50 (54%)	0	14/50 (28%)	0	0	3/50 (6%)	0	1/50 (2%)

**Table 2:** The number of search results for each of the different terms that were related to the Patient Autonomy Act when searching with the Google search engine on 5 April 2019.

Category of Search Results	Root Domain	Advance Care Planning	Advance Decision	Advance Directive	Hospice Palliative Care	Refuse Treatment	Do Not Resuscitate	Extubation	Withdraw Life-Sustaining Treatments	Suicide Assistance	Euthanasia
Health and Welfare Department and its affiliated organizations	.mohw.gov.tw	1	2	1	0	0	0	0	0	0	0
other public hospitals and local health care institutions	.gov.tw	13	7	5	0	3	0	0	1	0	0
private hospitals	.org.tw∩ hospital	14	8	5	0	2	0	0	0	0	0
non-governmental organizations	.org.tw∩ organization	6	5	5	0	0	0	0	1	0	0
non-governmental websites		2	12	6	0	8	0	0	1	0	0
electronic news or electronic magazines		10	11	8	0	3	0	0	0	0	1
Blogs	pixnet.net ∪ blogspot.com	0	0	0	0	1	0	0	0	0	0
Facebook pages		0	0	0	0	0	0	0	0	0	0
YouTube pages		0	1	1	0	2	0	0	0	0	0
explanations of laws and regulations from law firms		3	2	0	0	1	0	0	0	0	0
patient autonomy research centers	.parc.tw	1	1	0	0	2	0	0	0	0	0
Wikipedia	wikipedia.org	1	1	0	0	0	0	0	0	0	0
Total		50/50 (100%)	50/50 (100%)	31/50 (62%)	0	22/50 (44%)	0	0	3/50 (6%)	0	1 (2%)
